# Access to citizenship in selected European countries: comparative perspectives on legal regimes and intensity of acquisitions

**DOI:** 10.3389/fsoc.2026.1805881

**Published:** 2026-10-06

**Authors:** Maria Vittoria Forte, Salvatore Strozza

**Affiliations:** 1 Department of Political Sciences, University of Naples Federico II, Naples, Italy; 2 Institute for Research on Population and Social Policies (IRPPS), National Research Council (CNR), Rome, Italy

**Keywords:** citizenship laws, cross-country comparison, European countries, international migrants, migration policy, naturalization rates

## Abstract

Citizenship combines a formal legal bond with a State and a sense of belonging to a political community—a tension made especially visible by long-term migration, which produces populations integrated in society yet often excluded from full citizenship. Naturalization is where this tension plays out, shaped by national laws that vary widely across countries. This study examines how citizenship policy relates to acquisition outcomes across eighteen European countries (former EU-15 plus Poland, Norway, and Switzerland) over 2009–2023, addressing three open questions: the temporal stability of this relationship, the role of foreign population composition, and appropriate measurement strategies. Drawing on the comparative literature and the GLOBALCIT Citizenship Law Dataset, we analyze citizenship acquisition pathways (birthright, childhood provisions, ordinary and special naturalization) across the eighteen countries, and review existing policy indices to justify our selection of the MIPEX Citizenship Indicator as the policy-output measure. Outcomes are operationalized through citizenship acquisition rates (CARs) from Eurostat data, disaggregated by age (under 20 vs 20 and over) and citizenship status (EU vs non-EU), plus a standardized rate (SCAR). OLS regressions test the policy–outcome relationship across three five-year periods (2009–2013, 2014–2018, 2019–2023) and the 2014–2023 decade, controlling for EU citizens’ share, minors’ share, and refugee impact. MIPEX significantly predicts crude CARs in 2009–2018, but explanatory power drops sharply in 2019–2023. Only in some periods is the share of EU citizens among foreigners significantly and negatively associated, and the refugee impact positively associated, with CARs. The share of minors, instead, shows no significant effect in any period. Non-EU nationals and persons under 20 consistently naturalize at higher rates than EU nationals and adults, respectively, with only a few country-specific exceptions. Standardized rates (SCAR) improve model fit over crude CARs, especially in earlier periods. Formal legal inclusiveness matters but is a time-bound, moderately powerful predictor, mediated by population composition and weakening over time—likely reflecting unmeasured factors like implementation practices and category-specific pathways. Disaggregated and standardized CARs offer more robust tools for cross-national comparison than crude naturalization rates alone.

## 1 Introduction

Citizenship is a concept that has accompanied the history of political thought, assuming different meanings across epochs while retaining certain constant features (Paparusso and de Wenden, 2025). On the one hand, it has always implied a stable legal bond of subjection to a political authority; on the other, it has signified belonging to and participation in a political community—both prerequisites for the enjoyment of rights and prerogatives reserved to citizens and denied to foreigners. At the same time, citizenship cannot nowadays be reduced either to a purely political membership or to a merely legal status. As longstanding scholarship has shown, citizenship is a multidimensional and fluid concept, shaped by civil, political, and social rights (Marshall, 1950). It combines overlapping but not necessarily fully coincident dimensions of legal status, social recognition, political participation, membership, and belonging, and is therefore best understood as a dynamic configuration rather than a fixed or homogeneous condition (Bloemraad et al., 2008; Bosniak, 2006; Bloemraad and Sheares, 2017). Citizenship distinguishes between insiders and outsiders, and thus performs a selective function: as Brubaker (1992) has noted, it can be understood as a powerful instrument of social closure, both externally—by drawing a line between citizens and non-citizens at the border—and internally, by differentiating the legal status and rights of citizens from those accorded to foreign residents. This dual nature has become particularly salient in the contemporary context (Balibar, 2015), as globalization and the consolidation of universal human rights have increasingly blurred the boundaries between citizens and non-citizens, prompting renewed debates on the role of citizenship in plural and mobile societies (Bloemraad et al., 2008; Baraggia, 2017).

International migration has brought this tension to the forefront by challenging nationally bounded understandings of citizenship and unsettling the assumed congruence between nationality, residence, rights, belonging, and political participation. The long-term settlement of foreign populations has produced societies in which many individuals are socially and economically embedded, while remaining formally excluded from full political membership. Migration has therefore both reshaped the boundaries between citizens and non-citizens and enriched scholarly debates on citizenship by exposing the limits of models grounded exclusively in national membership. Yet, despite these transformations, access to citizenship continues to be regulated primarily through national legal regimes. From this perspective, naturalization constitutes a key institutional site where the tension between national citizenship and migratory societies becomes empirically observable. Against this background, the formal rules governing access to citizenship for foreign residents have attracted sustained scholarly attention, because they are central to understanding how States respond to the membership claims generated by migration. These rules vary markedly across countries, and have direct consequences for immigrant integration, political inclusion, and the reproduction of boundaries between citizens and non-citizens^1^ (Barbulescu et al., 2023; Milani, 2023).

Within comparative migration research, a substantial literature has examined both the legal design of citizenship regimes and the extent to which more inclusive citizenship laws are associated with higher levels of naturalization (Weil, 2001; Janoski, 2010; Vink, 2017; Strozza et al., 2021; Dicosola, 2022; Van der Baaren and Vink, 2024). Yet three issues remain insufficiently resolved: whether conventional naturalization rates are the most appropriate measure of citizenship acquisition outcomes for cross-country comparison, whether the policy-outcome relationship is stable over time, and whether it is distorted by the demographic composition of the foreign population.

This study contributes to this literature by introducing multiple operationalizations of citizenship acquisition rates (CARs) and by testing how formal policy inclusiveness—measured through the Access to Nationality strand of the Migrant Integration Policy Index (MIPEX), hereafter referred to as the ‘MIPEX Citizenship Indicator’—translates into acquisition patterns across eighteen European countries—comprising the EU-15 plus Poland, Norway, and Switzerland—over a fifteen-year period (2009–2023), while accounting for compositional differences by sex, age, and citizenship status (EU versus non-EU nationals). By doing so, the article contributes to the broader debate on the migration-citizenship nexus by showing how naturalization outcomes can be used to observe, comparatively and empirically, the ways in which national citizenship regimes respond to migration-driven claims to membership.

Three main research questions guide our analysis. First, what is the magnitude and temporal stability of the relationship between citizenship policy inclusiveness—as measured by the MIPEX Citizenship Indicator—and citizenship acquisition rates across the eighteen countries and over the 2009–2023 period? Second, do compositional characteristics of the resident foreign population—specifically, the proportion of EU nationals, recognized refugees, and minors—serve as explanatory variables for variation in acquisition rates across countries and over time? Third, do acquisition rates vary significantly by age (persons under 20 versus adults aged 20 and over) and citizenship status (EU versus non-EU nationals), and does using standardized rates that control for these compositional differences strengthen the policy-outcome relationship?

To address these questions, the article proceeds as follows. Section 2 documents the multi-dimensionality of European citizenship acquisition regimes, reviews the development of citizenship policy indices and their limitations, justifies the adoption of the MIPEX Citizenship Indicator, and develops the hypotheses on the relationship between policy outputs and citizenship acquisition outcomes. Section 3 presents data sources, proposed measures for the so-called CARs and analytical strategy. Section 4 reports the empirical results on the relation between MIPEX Citizenship Indicator and CARs. Section 5 discusses the findings, their implications, and limitations.

## 2 Citizenship policies, their indices and naturalization outcomes: research perspectives

Comparative scholarship has consistently documented significant transformations in European citizenship regimes since the 1980s (Howard, 2009). In response to migration-driven social and demographic change, Western European States progressively introduced conditional and double *ius soli* provisions, producing increasingly hybrid regimes (Vink and De Groot, 2010; De Groot and Vonk, 2018). This development has largely superseded the classical civic–ethnic typology (Brubaker, 1992), even though descent-based rules remained the structural core of citizenship at birth (Bertocchi and Strozzi, 2020). From the early 2000s onwards, this evolution has followed a dual and apparently contradictory trajectory (Dicosola, 2022): on the one hand, several traditionally more restrictive States—most notably Germany in 2000, and Portugal in 2006—reduced minimum residence requirements and expanded tolerance for dual citizenship (Montanari, 2012); on the other, the same period witnessed the widespread introduction of substantive integration requirements as preconditions for ordinary naturalization.^2^ More recently, these two tendencies have pulled in increasingly divergent directions, as migration pressures and security concerns have led several States to reinforce revocation provisions and tighten substantive admission conditions, while others have continued along a liberalizing path (Dicosola, 2022). The following subsection documents the principal structural features of this fragmented landscape, as a basis for understanding both the choices made by citizenship policy indices and the empirical analysis in Sections 3 and 4.

### 2.1 The multi-dimensionality of citizenship acquisition regimes

Citizenship acquisition regimes in European countries are shaped by several interacting legal dimensions, including rules governing acquisition at birth and in childhood, conditions for ordinary naturalization, facilitated pathways for specific categories of applicants, and provisions on dual citizenship (Vink and Bauböck, 2013). These dimensions correspond to the main modes of citizenship acquisition documented in large-scale comparative datasets such as the GLOBALCIT Citizenship Law Dataset (Vink et al., 2021), whose results confirm their centrality across European legal systems. As regards birthright and childhood-based pathways, while *ius sanguinis* remains the dominant principle across the eighteen countries, territorial birth for children of non-citizens ranges from complete absence—as in Austria, Denmark, Finland, Italy, Norway, Poland, Sweden, and Switzerland—to conditional *ius soli* linked to parental residence requirements, as in Germany, Ireland, Portugal, and the United Kingdom, to double *ius soli* provisions, as in Belgium, France, Greece, Luxembourg, Portugal, and Spain, where citizenship is attributed to a child born in the country if at least one parent was also born there, albeit with varying additional conditions (Dicosola, 2022). Beyond birth, most countries provide further childhood-based facilitation through weak *ius soli* provisions, childhood socialization routes based on schooling or early arrival, and family-linked pathways tied to parental naturalization—either through co-acquisition alongside the naturalizing parent, available in almost all countries, or through derivative acquisition once a parent has already naturalized, as, among our selected European States, in Finland, Greece, the Netherlands, Norway, Sweden, and Switzerland (Goodman, 2010b). Looking at the weak *ius soli* provisions, Italy offers an illustrative example of the latent complexity of these provisions: children of foreign nationals born in Italy are not granted citizenship at birth, but may claim it through a declaration submitted within 1 year of reaching the age of majority, provided they have resided continuously in Italy from birth (Tintori, 2018).

Turning to ordinary naturalization—one of the dimensions most consistently captured by citizenship policy indices (see Section 2.2)—the relevant conditions encompass residence requirements, civic and economic integration conditions, good character clauses, and the acceptance of dual citizenship (Vink et al., 2025). However, the surface comparability of these dimensions conceals a degree of legal heterogeneity that synthetic indices tend to understate. Residence requirements illustrate this well: nominal thresholds range from 5 years in most Western European countries to 9 or 10 years in Austria, Denmark, Italy, Spain, and Switzerland, yet what counts as qualifying residence varies as much as the duration itself. In several countries, naturalization requires prior permanent residence, making effective waiting times systematically longer than nominal thresholds suggest; elsewhere, rules on continuous presence and tolerated absences introduce further cross-national variation that is rarely reflected in summary scores. Civic and economic integration conditions govern whether applicants can demonstrate the degree of social membership that States increasingly treat as a precondition for citizenship, and they have become structurally significant dimensions (Goodman, 2010a; Paparusso, 2016). Language proficiency requirements vary not only in the required level—B1 in Austria, Germany, Italy, and Poland; A2 in Portugal; with France transitioning from B1 to B2 as of 2026—but also in the certifying bodies recognized, the categories of exemption, and whether schooling in the country substitutes for a formal test. On the other hand, economic self-sufficiency conditions ranges from proof of stable income—as in the cases of Austria, Greece, and Italy—to non-reliance on social benefits in Denmark, Finland, and Germany. Finally, good character clauses differ substantially in the types of infraction that trigger disqualification, the temporal duration of their effects, and the role of security screenings, which in some countries operate as a *de facto* additional barrier independently of standard criminal record checks.

The acceptance or rejection of dual citizenship determines whether naturalization entails a genuine choice between legal memberships or can be pursued without severing prior national ties—a condition whose practical significance varies considerably across migrant populations depending on their country of origin, given that origin-country laws may themselves impose loss of nationality upon naturalization elsewhere (Vink, 2017; Van der Baaren and Vink, 2024). Looking at the eighteen selected countries, most of them do not require applicants for ordinary naturalization to renounce their previous nationality, with Austria, Germany, the Netherlands and Spain remaining—at least formally—the main exceptions.

Special facilitated routes, a dimension less consistently captured by synthetic indices, add a further layer of differentiation (Goodman, 2010b) even though they are particularly relevant when analyzing citizenship outcomes, since they are revealing remarkably popular in some EU countries (Kochenov and van den Brink, 2023). Spouses or registered partners of citizens benefit from reduced residence requirements in seventeen of the eighteen selected countries; recognized refugees have access to a dedicated naturalization track in thirteen countries, with residence requirements in same cases halved compared to the ordinary route (such as in Italy and Spain); nationals of specific countries—particularly EU citizens, Nordic nationals, or persons with ancestral or cultural ties—may benefit from further shortened residence periods or simplified declaration routes in several countries, including Germany, Greece, Italy, and Spain.

These legal patterns point to a dense and multi-layered regulatory landscape, which has fueled a large body of scholarship seeking to synthesize citizenship laws and policies into comparable indices (Vink and Bauböck, 2013; Vink, 2017). These indices vary in scope, level of detail, and temporal coverage, but share a common goal: translating complex legal regimes into analyzable indicators of citizenship policy inclusiveness and restrictiveness.

### 2.2 The development of citizenship policy indices

The comparative measurement of citizenship policies has expanded markedly since the late 1990s, shifting from the qualitative typologies that dominated earlier scholarship toward increasingly sophisticated quantitative indicators and legal datasets. Broadly speaking, this literature has developed along three main lines: qualitative and descriptive typologies, synthetic quantitative indices, and more granular legal datasets.

The first strand consists of qualitative and descriptive typologies. The most influential example is Brubaker’s (1992) distinction between civic-territorial and ethnic-descent models of citizenship, exemplified, respectively, by France and Germany. Building on this tradition, early comparative studies such as Waldrauch and Hofinger’s (1997) Legal Obstacles to Integration index and Weil’s (2001) comparative analysis of nationality laws adopted multidimensional coding schemes that considered citizenship acquisition at birth, ordinary naturalization, loss provisions, residence requirements, and dual citizenship tolerance. Although these studies provided important foundations for comparative research, their primary aim was descriptive rather than synthetic, as they did not assign explicit weights to individual dimensions or generate overall measures of citizenship policy inclusiveness.

A second strand developed during the 2000s with the emergence of synthetic quantitative indices designed to produce comparable country scores (Helbling, 2013; Goodman, 2015). Examples include Koopmans et al.’s (2005) indicators of nationality acquisition, Howard’s Citizenship Policy Index (Howard, 2009), and Janoski’s Barriers to Naturalization Index (Janoski, 2010). In particular, Koopmans et al. (2005) in their analysis of ‘configurations of citizenship’ in France, Germany, the Netherlands, the United Kingdom, and Switzerland at three time points (1980, 1990, 2002), developed indicators for ‘nationality acquisition’ covering residency years, welfare dependence prohibitions, facilitated access for second-generation immigrants, dual citizenship acceptance, absence of special rights for co-ethnics, and naturalization rates. Howard’s Citizenship Policy Index proposed a parsimonious 0–6 scale across three core dimensions—*ius soli* attribution, ordinary naturalization conditions like residency/language/spousal rules, and dual citizenship tolerance—and applied it to EU-15 countries in the 1980s and 2008; notably, it incorporated naturalization rates as a weighted corrective for *de jure–de facto* gaps. Janoski’s Barriers to Naturalization Index covered 18 OECD countries from 1970 to 2006, measuring restrictiveness via metrics such as minimum residency, economic self-sufficiency tests, good character requirements, and renunciation clauses.

Among these synthetic indices, the Migration Integration Policy Index (MIPEX) has become one of the most widely used instruments in comparative research. Introduced in its current form in 2007 by the Migration Policy Group and the British Council, MIPEX evaluates countries across several dimensions of migrant integration, including access to nationality. Its Citizenship Indicator is a composite measure ranging from 0 to 100 that captures the extent to which citizenship laws and procedures facilitate or hinder naturalization. The indicator aggregates detailed information on eligibility criteria, residence requirements, economic and civic integration conditions, criminal record checks, and tolerance of dual citizenship, with higher scores indicating more inclusive citizenship policies.

However, precisely because these indices are synthetic, they necessarily reduce a much denser legal landscape to a limited set of codable dimensions. In doing so, they tend to privilege ordinary naturalization and dual citizenship, while treating other relevant features—such as the legal meaning of residence, facilitated routes for specific categories of applicants, and facilitated pathways for second-generations [except for the indicators for ‘nationality acquisition’ by Koopmans et al. (2005)]—in a simplified or partial way. As a result, they are best understood as useful but incomplete synthetic measures of citizenship policy, rather than as exhaustive representations of the full architecture of access to citizenship regimes.

A third strand has shifted from producing summary scores to building granular legal datasets that systematically document the specific modes and conditions of citizenship law, enabling more direct cross-national comparison of individual legal provisions (Vink, 2017). The European Union Democracy Observatory on Citizenship (EUDO Citizenship) developed the CITLAW and CITIMP indicators (European Union Democracy Observatory on Citizenship, 2013): CITLAW systematically coded 27 distinct modes of citizenship acquisition and 11 provisions for loss of citizenship, while CITIMP provided a composite score based on five implementation facets, encompassing the entire administrative lifecycle. Since 2017, EUDO has evolved into the Global Citizenship Observatory (GLOBALCIT), which maintains and updates the GLOBALCIT Citizenship Law Dataset (Van der Baaren and Vink, 2021). Unlike CITLAW, which translated legal provisions into quantitative scores, the new Dataset adopts a categorical coding approach, systematically mapping the presence and main conditions of 28 modes of citizenship acquisition and 15 modes of citizenship loss across 190 countries, thereby facilitating the direct comparison of rules applicable to similar groups across national legal systems (Van der Baaren and Vink, 2021). It represents the only instrument in the EUDO/GLOBALCIT tradition available for the period from 2020 onwards.

Despite the proliferation of citizenship-specific indices, no single indicator has yet emerged as a universally accepted measure of citizenship policy inclusiveness.

### 2.3 Selection of the MIPEX Citizenship Indicator

For the purposes of this study, the MIPEX Citizenship Indicator has been selected as the principal measure of citizenship policy outputs. This choice reflects its combination of broad geographical and temporal coverage, availability for all eighteen countries included in the analysis through 2023, transparent coding procedures, and focus on the principal legal dimensions shaping access to citizenship. Alternative indices were also considered but set aside for different reasons: Howard’s CPI seemed less suitable for an output-oriented design, because it incorporates naturalization rates in the policy index and thus blurs the distinction between policy outputs and outcomes; CITLAW and CITIMP do not offer longitudinal and cross-national data for the period 2016 onwards; and GLOBALCIT provides qualitative coding rather than standardized quantitative country scores and covers only the period from 2020 onward.^3^ MIPEX therefore emerged as the most suitable instrument for linking policy outputs to naturalization rates in large comparative design in recent years, balancing recency, cross-national comparability, and analytical suitability.

However, this methodological choice entails certain trade-offs. The indicator is primarily a *de jure* measure and therefore cannot fully capture the ‘implementation gap’ (Huddleston and Falcke, 2020) between formal legal requirements and their practical application. Dimensions such as administrative implementation practices, bureaucratic discretion, processing times, fees, judicial review and local administrative cultures often remain invisible within the indicator’s composite scoring methodology. Moreover, the MIPEX Citizenship Indicator captures only part of the considerable legal complexity that characterizes the citizenship regimes (see Section 2.1). As a composite indicator, MIPEX necessarily simplifies cross-national differences in eligibility rules and procedural conditions for naturalization. It overlooks the fact that foreign residents are not all exposed to the same naturalization rules: minors, refugees, spouses of citizens, and nationals of specific countries may benefit from facilitated access to citizenship, thereby affecting acquisition rates independently of the level of policy inclusiveness measured by MIPEX (which does not fully capture the specificities of these facilitated pathways).

These limitations, however, do not invalidate the analysis; rather, they suggest that MIPEX-based results should be interpreted with due consideration for the legal heterogeneity of citizenship acquisition pathways. Future research could further strengthen comparative analyses by incorporating more detailed indicators capturing facilitated naturalization pathways and group-specific access to citizenship, as well as, reasonably, qualitative measures of administrative implementation.

### 2.4 Policy-outcome relationships and hypotheses

Research on citizenship regimes has long focused on comparing policy outputs across countries and over time, examining both the determinants of citizenship regimes and their consequences for one of their most direct outcomes: the acquisition of citizenship. A broad body of scholarship suggests that more inclusive citizenship policies facilitate citizenship acquisition. Yet, while the expectation of a positive association between policy inclusiveness and citizenship acquisition is well established, the strength, stability, and cross-national comparability of this relationship remain far less clear. Differences in eligibility structures, migrant population composition, policy implementation, and the limitations of available outcome indicators make it difficult to determine whether observed variation in citizenship acquisition reflects policy inclusiveness itself or broader contextual factors.

Assessing this relationship comparatively is therefore challenging. On the policy side, formal legal provisions may not be implemented uniformly, as administrative practices, bureaucratic discretion, and institutional capacity can mediate the effects of citizenship laws. On the outcome side, the demographic composition of foreign populations, variation in eligibility, and measurement choices all influence observed levels of citizenship acquisition. As a result, similar levels of policy inclusiveness may produce different citizenship acquisition outcomes across countries, while similar acquisition rates may emerge under substantially different policy regimes.

A substantial body of research has shown that legal requirements—including eligibility criteria, residence requirements, language and integration tests, economic conditions, and dual citizenship acceptance, dimensions generally operationalized across indices such as MIPEX—shape citizenship acquisition, although their effects may differ across countries, over time, and across categories of foreign residents (Janoski, 2010; Reichel, 2011; Dronkers and Vink, 2012; Vink, 2017; Huddleston and Falcke, 2020). However, since citizenship acquisition pathways are broader and more complex than policy indices can fully capture (see Sections 2.1 and 2.2), and because migrant populations vary significantly across countries, naturalization rates likewise differ greatly and cannot be interpreted as straightforward reflections of policy inclusiveness as synthesized by existing indices alone.

Recent individual-level research further confirms this complexity. Labussière and Vink (2020), for instance, link policy and outcomes through individual-level register data, showing that different citizenship reforms may have different effects: some restrictions, such as dual citizenship limits, may deter naturalization, whereas more ‘thick’ reforms, such as mandatory civic integration, may mostly delay it. However, this approach remains limited to a small number of countries due to data availability constraints and does not readily allow for cross-country comparison.

Two further considerations warrant attention: first, the role of policy implementation, since administrative practices and discretionary application mediate the effects of formal legal provisions (Van der Baaren, 2024); and second, the measurement of citizenship policy outcomes, since classic naturalization rates (acquisitions in a given year as share of initial foreign population) are principally criticized for their lack of probabilistic interpretation and for their heightened sensitivity to fluctuations in immigration inflows that perturb the denominator (Perrin, 2006; Helbling, 2011; Reichel, 2011). Alternative measures include rates based on eligible populations (Reichel, 2011) or cohort-based rates that yield genuine naturalization probabilities (Perrin, 2006), and rejection rates (Helbling, 2011), but they also face data scarcities and methodological caveats (Reichel, 2011; Vink, 2011). Reichel’s EU-15 analysis (2002–2007) illustrates these limitations, disclosing only weak covariation between MIPEX citizenship policy outputs (2006) and contemporaneous average naturalization rates—a linkage that evaporates entirely when Sweden is excluded—thereby underscoring the inadequacy of unadjusted and general naturalization rates for evaluating citizenship policies’ outcomes. Still, as Reichel (2011, p. 8) advanced, those general naturalization rates retain value as ‘general demographic indicator for the population acquiring citizenship’, especially under relatively constant immigration flows, whilst the alternatives proposed in the literature present much consistent issues of data availability and cross-country comparability. Given the persistent limitations affecting alternative indicators, and the need to maximize temporal and cross-national comparability, this study employs citizenship acquisition rates constructed according to the procedure described in Section 3 as its principal measure of citizenship policy outcomes.

Maintaining the distinction between policy outputs (operationalized through the MIPEX Citizenship Indicator) and outcomes (operationalized through the citizenship acquisition rate, explained in Section 3) is essential to obtain a complete picture for normative evaluation and empirical purposes (Helbling, 2011; Reichel, 2011; Vink, 2011, 2017). Accordingly, the contribution of this study is not simply to verify whether more inclusive citizenship policies are associated with higher citizenship acquisition rates. Rather, building on the theoretical framework outlined above and the heterogeneity of citizenship acquisition pathways, the study examines how stable and comparable the relationship between citizenship policy outputs and citizenship acquisition outcomes remains across eighteen European countries over the period 2009–2023, while accounting for differences in the composition of the foreign population and for the limitations of available outcome indicators.

Particular attention is devoted to the composition of the foreign resident population, since some categories—including minors, spouses, refugees, and persons with specific cultural, historical, or national ties—may benefit from facilitated pathways to citizenship (see Section 2.1) and therefore display acquisition patterns that are only partially explained by the general inclusiveness of national citizenship regimes. The analysis therefore evaluates whether and to what extent accounting for these compositional differences improves the explanatory power and cross-national comparability of the policy–outcome relationship.

Drawing on the research questions posed in the introduction, three sets of hypotheses guide the empirical analysis:

*H1 (Policy-Outcome Relationships):* More inclusive citizenship policies, as measured by higher MIPEX Citizenship Indicator scores, are positively associated with higher CARs across countries and over time.

*H2.a (EU citizens Composition Effect):* A higher proportion of EU citizens within the foreign resident population is negatively associated with CARs. EU nationals, already enjoying intra-EU mobility rights and freedom of residence, face reduced incentives to acquire host-country citizenship.

*H2.b (Minors Composition Effects):* A higher proportion of minors among foreign residents is positively associated with CARs. Minors may benefit from more favorable provisions since they may access citizenship through facilitated pathways—including conditional birthright rules, post-birth tracks based on childhood residence or socialization, and family-linked mechanisms (see Section 2.1)—which makes them structurally more likely to acquire citizenship than adults.

*H2.c (Refugee Composition Effect):* A higher refugee-to-population ratio is positively associated with CARs, since refugees may benefit from more favorable naturalization provisions, such as reduced residence period required for naturalization. Moreover, the presence and recognition of refugee populations may serve as a broad proxy for institutional and administrative infrastructures supportive of immigrant integration, since it captures the volume of asylum applications alongside the State’s willingness and capability to manage these arrivals and grant protection^4^, which may in turn signal an underlying infrastructure that facilitates the integration of foreign nationals more broadly.^5^

*H3.a (Disaggregation effects):* The association between the MIPEX Citizenship Indicator and CARs is stronger when CARs are disaggregated by relevant sub-populations, particularly age group and EU/non-EU foreign nationals.

*H3.b (Targeted Measurement Effects):* The association between the MIPEX Citizenship Indicator and CARs is stronger for CARs calculated on target populations, especially non-EU nationals and adults.

*H3.c (Compositional Controls’ Effects):* The association between the MIPEX Citizenship Indicator and CARs is stronger when models control for the demographic composition of the foreign resident population, particularly the shares of minors and EU nationals.

## 3 Materials, measures and methods

### 3.1 Data sources

To empirically test the hypotheses advanced, this contribution drew on EUROSTAT data concerning citizenship acquisitions^6^ and foreign resident populations in the eighteen selected European countries for the period 2009–2023, always disaggregated by sex. Where data were missing, we used data from countries’ National Statistical Institutes, after verifying their consistency with EUROSTAT disseminations. The eighteen countries comprise the EU-15 plus Poland, Norway and Switzerland. The selection of these countries rests on two grounds. First, the selected countries have consistently maintained significant foreign populations, both in absolute and relative terms, throughout the 2009–2024 period.^7^ On this basis, we excluded the newer Member States of Central and Eastern Europe, with the exception of Poland, which was included because of its demographic size and its growing relevance in recent years as an immigration country. Second, we included Norway and Switzerland alongside EU Member States because, despite their non-EU membership, they are deeply integrated into the EU migration space.^8^

Missing values in citizenship acquisitions and foreign populations by age group (considering only persons under 20 and those aged 20 and over) and, more frequently, by citizenship (foreigners with citizenship of EU and non-EU countries)^9^ were imputed using distributions from adjacent years where information was available. With regard to the population, we supplemented annual population data with data from the 2011 and 2021 demographic censuses, available for all countries except the United Kingdom for the most recent survey.^10^

### 3.2 Proposed measures of citizenship acquisitions

To measure citizenship acquisition intensity^11^ we calculated the crude citizenship acquisition rate (CAR) as the ratio between the number of citizenship acquisitions and the person-years of foreign residents. These rates were calculated for each individual year and for the three five-year periods 2009–2013, 2014–2018 and 2019–2023, as well as for the last decade (2014–2023). Rate values express the average annual number of citizenship acquisitions per 1,000 foreign residents. The calculation formula for a period of *n* years is:


$$\mathrm{Ct}-t+\mathrm{nAR}=\frac{\mathrm{Ct}-t+\mathrm{nA}}{\sum_{i=0}^{n}(\text{Ft}-1+\mathrm{iP}+\text{Ft}+\mathrm{iP})/2}\cdot 1,000$$

where Ct−t+nA indicates the number of citizenship acquisitions in the period *t*, *t* + *n*, Ft−1P the number of foreign residents at the end of year *t*−1, and Ft+nP the number of foreign residents at the end of year *t* + *n*. The denominator of the ratio represents an estimate of the person-years lived by foreign residents in the period under consideration (*t*–*t* + *n*). In substance, we summed the arithmetic mean (semi-sum) of residents in each year composing the period. This estimation criterion appears most appropriate given that year-to-year changes in foreign resident numbers may be substantial.

The proposed indicator differs from the ‘classic’ naturalization rate—i.e., the one commonly employed in the literature (Helbling, 2011, p. 3) and calculated by EUROSTAT—in how the denominator is constructed. Rather than using the stock of foreign residents at the beginning of the period, it calculates the total person-years lived by foreign residents during the period under consideration. In this way, the resulting rate expresses the average annual number of citizenship acquisitions per 1,000 foreign nationals.

In the numerator, we considered all citizenship acquisitions, regardless of the mode of acquisition, thus including not only ordinary naturalization but also all other citizenship acquisition pathways. For this reason, the term CAR appears to be more appropriate than naturalization rate, which refers specifically to the ordinary naturalization pathway.^12^

However, this CAR remains a crude rate (so crude CAR) expressing only relative frequency, without accounting for differences in foreign population composition. For this reason, we also calculated age-specific and citizenship-specific CARs. In this way, the analysis captures some of the compositional differences relevant to first- and second-generation trajectories, although age could serve here only as a proxy. We computed CARs for foreign persons under 20 and for those aged 20 and over, as well as for foreigners with EU citizenship and those with non-EU citizenship, always disaggregated by sex. Calculation methods are equivalent to those in the formula above, considering only the numerator and denominator relating to the population category under examination. This enables verification of significant differences in citizenship acquisition intensities among different foreign population categories, testing the hypothesis named H3.a.

We also proposed a sex-age-citizenship-standardized CAR (SCAR) as an alternative measure that controls for compositional differences according to these three characteristics of foreign resident populations across the examined countries. We obtained the standardized rate as a weighted average of sex-age-citizenship-specific CARs, using as weights the proportion of the foreign population according to these three characteristics observed in 2011 across the eighteen selected countries. In formula:


$$\text{SCAR}=\sum_{s}\sum_{a}\sum_{c}\mathrm{CsA}R_{a}^{c}\cdot \bar{\mathrm{FP}}s_{a}^{c}$$

where $\mathrm{CsA}R_{a}^{c}$ are the sex(*s*)-age(*a*)-citizenship(*c*)-specific CARs and $\bar{\mathrm{FP}}s_{a}^{c}\;$ the proportion of foreign population according to the three characteristics considered, with the sum across sex, age and citizenship equal to one.

### 3.3 Method of analysis

Each of the different rates calculated in the above-mentioned ways constitutes a dependent variable in some ordinary least squares (OLS) regression models that adopt among the explanatory variables at least the MIPEX Citizenship Indicator and use the eighteen selected European countries as statistical units.^13^ We applied these models exclusively to data for the three five-year periods (2009–2013, 2014–2018 and 2019–2023) and for the last decade (2014–2023). In this way, we worked with average data for a period sufficiently broad to reduce annual fluctuations.

To verify H1, an initial application considered the crude CAR as dependent variable and the MIPEX Citizenship Indicator (for further details on the construction methods, strengths and limitations of this thematic indicator, see Section 2) exclusively as explanatory variable. To verify H2.a, H2.b, and H2.c subsequent applications added three explanatory variables to the MIPEX Citizenship Indicator: percentage of under 18 s (minors) among foreign residents; percentage of EU citizens among foreign residents; refugees per 1,000 inhabitants (national and foreign residents). While the first two additional indicators derive from the EUROSTAT data already described, the last indicator was calculated using United Nations High Commissioner for Refugees (UNHCR) data for the numerator and EUROSTAT population data for the denominator. Importantly, the first two explanatory variables refer to the composition of the foreign population, whereas the refugee variable is included as a separate contextual predictor and should not be conflated with the compositional indicators of the foreign population, which are limited to age and citizenship status (EU vs. non-EU nationals).

We carried out a second block of OLS multiple regressions to verify H3.b and H3.c. We considered as dependent variables each of the four specific rates: CARs for foreign EU citizens, non-EU citizens, those under 20 years, and those aged 20 and over. The SCAR serves as the fifth dependent variable. All these regressions (20 in total, given by the product of the 5 dependent variables times the 4 multi-year periods considered), employed the same two independent variables: MIPEX Citizenship Indicator and refugees per 1,000 inhabitants.

## 4 Results

### 4.1 Crude CARs and their correlation with laws and foreign characteristics

Over the period 2009–2023, citizenship acquisitions in the selected countries totaled more than 14 million, of which approximately 2.5 million occurred in the United Kingdom, over 2 million in Spain, around 1.9 million in Italy, more than 1.8 million in Germany, and nearly 1.7 million in France (Table 1). These are the countries with the largest demographic size, all of which now have a long history of immigration, although Italy’s and Spain’s immigration history is relatively more recent. These latter two countries have witnessed an increase in the number of citizenship acquisitions over the last or the last two five-year periods, whereas in France acquisitions have decreased slightly, and in the other two countries (Germany and the United Kingdom) they have remained substantially stable.

**Table 1:** Acquisitions of citizenship by foreign citizens in 18 European selected countries period 2009-2023.

Country	Label	Number of acquisitions of citizenship (in thousand)				Citizenship acquisition rates (per 1,000 foreigners)		
		2009–13	2014–18	2019–23	2009–23	2009–13	2014–18	2019–23
Belgium	BE	170.6	151.3	217.4	539.4	29.6	22.8	29.0
Denmark	DK	20.5	41.6	23.8	85.9	11.6	17.8	8.4
Germany	DE	536.7	565.8	739.6	1,842.1	16.1	13.0	13.6
Ireland	IE	71.0	61.1	52.9	185.0	25.6	21.8	15.5
Greece	GR	93.7	130.2	65.8	289.7	20.5	31.8	15.9
Spain	ES	637.8	628.4	791.0	2,057.3	24.5	27.8	28.3
France	FR	587.0	562.7	538.5	1,688.1	29.8	24.3	19.9
Italy	IT	347.6	768.6	807.5	1,923.7	16.5	31.7	31.7
Luxembourg	LU	19.0	21.6	26.4	67.0	16.7	15.8	17.4
Netherlands	NL	141.5	144.6	263.7	549.7	41.3	32.7	42.0
Austria	AT	35.2	42.7	51.8	129.7	7.5	6.7	6.6
Poland	PL	16.6	21.1	42.2	79.8	39.5	23.3	20.6
Portugal	PT	115.5	106.0	115.6	337.0	54.6	55.9	35.2
Finland	FI	30.3	47.0	46.5	123.8	34.8	40.2	31.6
Sweden	SE	199.0	286.6	393.7	879.3	63.5	71.0	89.1
Norway	NO	63.7	73.9	150.7	288.3	32.7	27.3	50.0
Switzerland	CH	186.3	203.9	194.9	585.2	20.8	19.8	17.4
United Kingdom	UK	977.4	673.1	862.3	2,512.8	41.6	23.2	28.5

Sources: own elaborations on data collected by EUROSTAT, supplemented where necessary with data from national statistical institutes.

Absolute values in thousands and rates per 1,000 foreigners.

When moving from absolute values to relative figures, the picture changed significantly. Based on the crude CAR values, none of the five above-mentioned countries ranked in the top five positions in the period 2009–2013, while in the two subsequent five-year periods only Italy entered the top five, ranking fifth. Across all three periods, Sweden was the country with the highest values (between just under 65 and almost 90 citizenship acquisitions on average per year per 1,000 foreign residents), followed in the period 2009–2013 by Portugal and the United Kingdom, in the period 2014–2018 by Portugal and Finland, and in the period 2019–2023 by Norway and the Netherlands. Austria instead registered the lowest values (less than 10 per 1,000) in three periods, alongside Germany, Denmark, and Luxembourg.

The crude CARs calculated year-by-year (Figure 1) confirmed some underlying trends, even though fluctuations were not negligible and suggested conducting the analysis over multi-year periods.^14^ Nevertheless, it is worth noting how the relative frequency of citizenship acquisitions was consistently low in the cases of Austria, Germany and Luxembourg; high and increasing in Sweden and Norway; and high but recently declining in Portugal, probably due to substantial recent immigration. Among the countries that registered some peaks in crude CAR values, the cases of Spain and Italy were particularly noteworthy—countries of relatively recent immigration with relatively restrictive legislations on access to citizenship.

**Figure 1:**
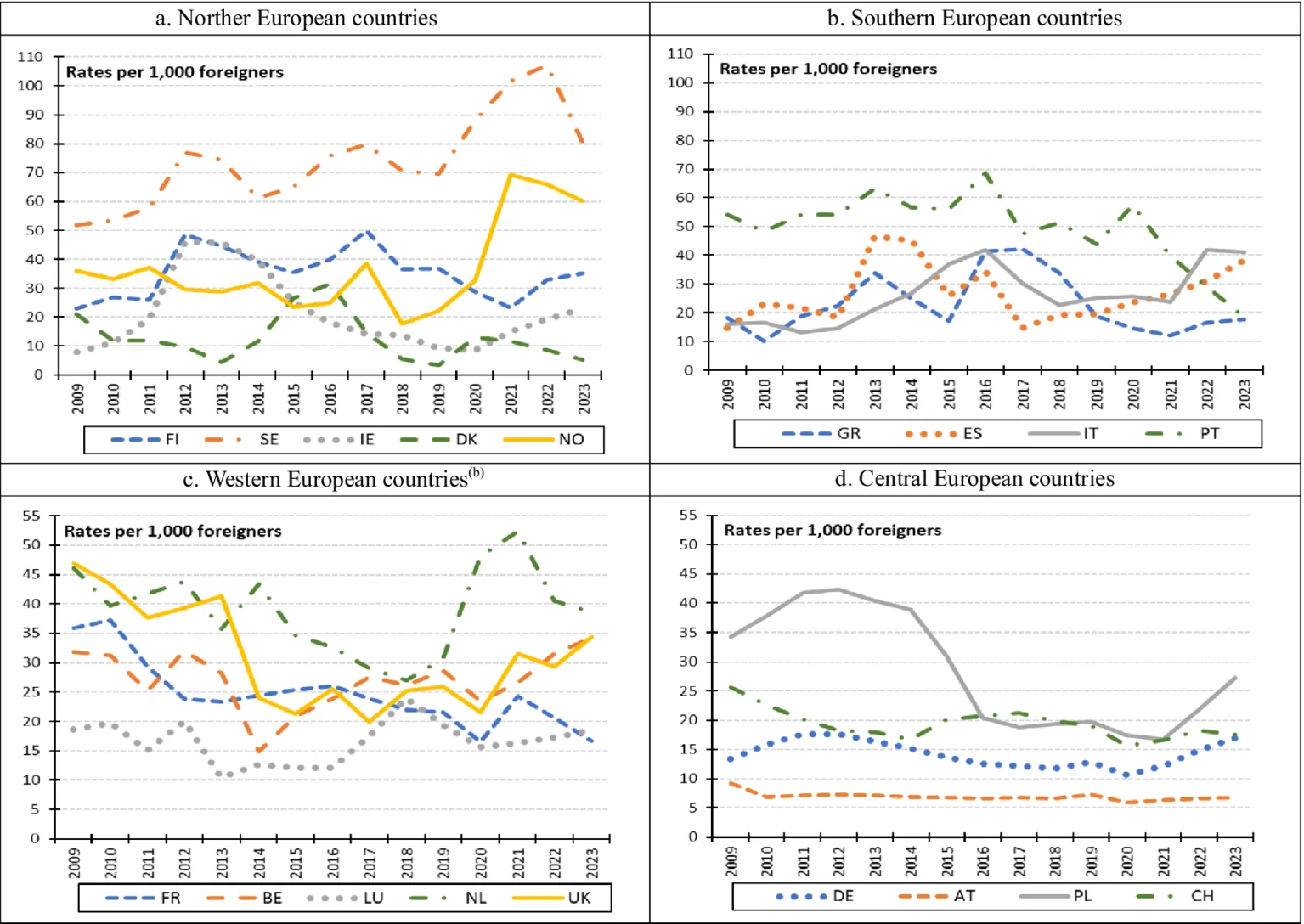
Citizenship acquisition rates in 18 European selected countries^(a)^: Northern European countries **(a)**, Southern European countries **(b)**, Western European countries **(c)**, Central European countries **(d)**. Period 2009–2023 (rates per 1,000 foreigners). ^(a)^See Table 1 for the correspondence between labels and countries. ^(b)^For convenience, the UK was included in this group. Sources: see Table 1.

As outlined in Section 2, the MIPEX Citizenship Indicator ranges from 0 (critically unfavorable) to 100 (favorable), with intermediate categories of slightly unfavorable (21–40), halfway favorable (41–59), slightly favorable (60–79), and favorable (80–100). Austria remained below 20 throughout all time periods, followed by Spain and Switzerland, which consistently scored below 40. Italy fell below 40 in two sub-periods, and Denmark and Greece in the first and last periods examined, respectively. At the upper end, Sweden scored in the favorable range in all periods, while Portugal did so from 2019 onward. Belgium, Finland, France, Ireland, Luxembourg, and the UK cluster in the slightly favorable range, while the remaining countries fall in the halfway favorable range (Figure 2).

**Figure 2:**
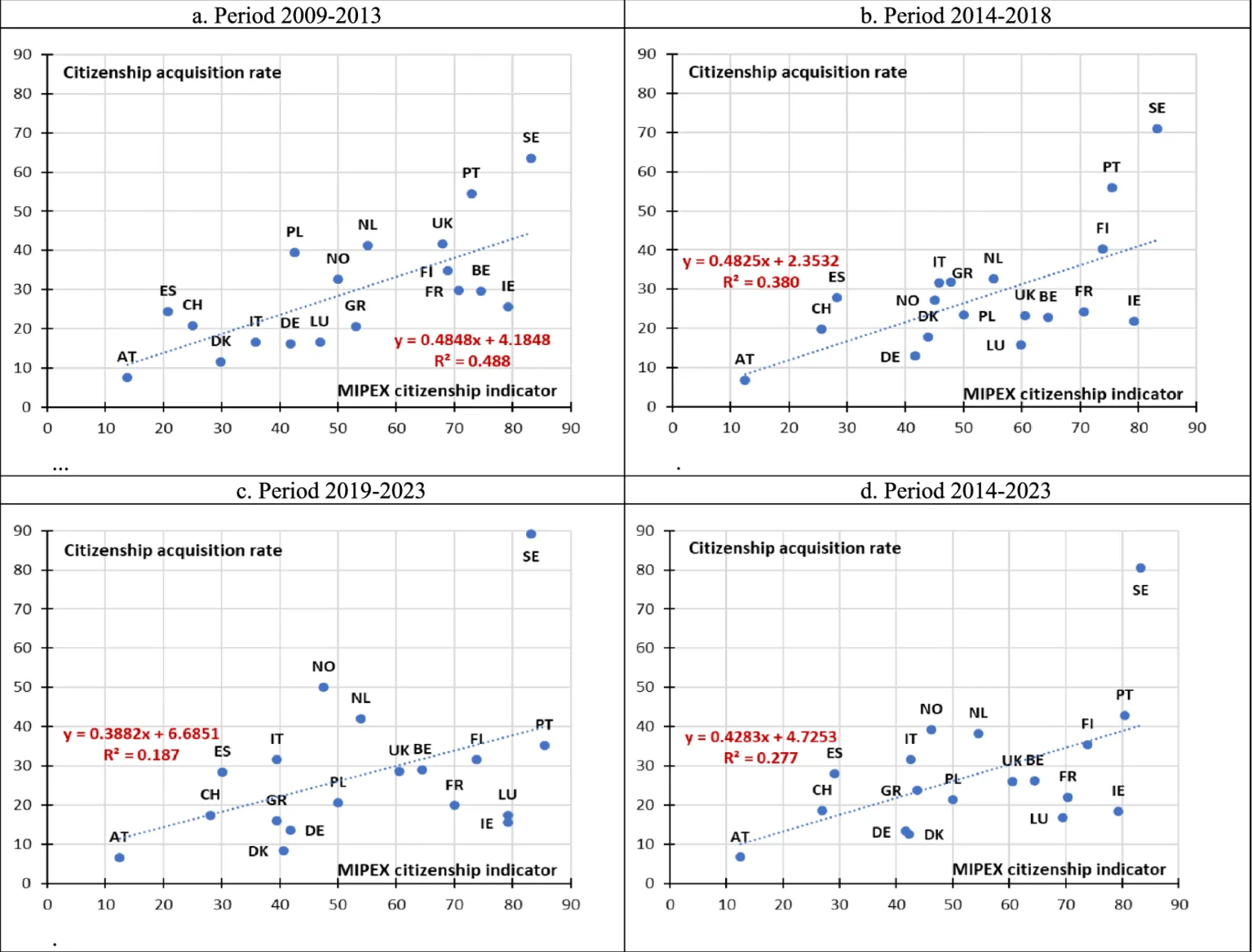
Correlation between MIPEX Citizenship Indicator (0–100) and citizenship acquisition rates (per 1,000 foreigners) considering 18 European selected countries^(a)^. Periods 2009–2013 **(a)**, 2014–2018 **(b)**, 2019–2023 **(c)**, and 2014–2023 **(d)**. ^(a)^See Table 1 for the correspondence between labels and countries. Sources: see Table 1.

The relationship between the MIPEX Citizenship Indicator and crude CAR values appeared evident in 2009–2013 but weakened substantially in subsequent periods (Figure 2). The explanatory capacity of the MIPEX Citizenship Indicator diminished consistently from a period to another: the variability explained by the simple linear regression model, measured by the coefficient of determination (*R*^2^), declined from nearly 50% in the first period to less than 40% in 2014–2018, falling to 18.7% in 2019–2023. Over the entire decade 2014–2023, *R*^2^ settled around 28%. Furthermore, the linear relationship not only weakened over time, but also appeared increasingly influenced by the case of Sweden, which Reichel (2011) had already shown to be an outlier, with crude CAR values markedly higher than those expected on the basis of the linear relationship, particularly in the last five-year period. Values clearly above those expected according to the linear model were observed for Portugal, the Netherlands, Poland and Spain in the period 2014–2018; for Portugal, Spain and Italy in the period 2014–2023; and for Norway, the Netherlands, Spain and Italy in the period 2019–2023. Conversely, across all three periods, Germany, Ireland and Luxembourg presented crude CAR values lower than those expected according to the linear model. France, particularly in the last two five-year periods, and Denmark, particularly in the first two periods, were in the same situation.

The crude CAR values in the selected countries also depend on characteristics of the foreign population residing there, since certain categories of foreigners may be more or less favored in acquiring citizenship. Therefore, cross-country differences in crude CAR may partly reflect compositional differences in foreign populations. For this reason, we supplemented the MIPEX Citizenship Indicator with additional explanatory variables capturing key characteristics of the foreign resident population in multiple linear regressions.

The independent variables added were the percentage of minors (under 18 years), EU citizens among foreigners, and refugee impact on the total population (per 1,000 inhabitants). Adding these variables enhanced the models’ explanatory capacity in three of the four periods (the first two five-year periods and the decade 2014–2023), as documented by the adjusted *R*^2^ values (in the simple linear regressions of Figure 2 they are: 0.456, 0.342, 0.136, and 0.231), without presenting significant multicollinearity situations (Table 2). Indeed, tolerance values remained close to one and the Variance Inflation Factor (VIF) values stayed markedly below 5.

**Table 2:** Results of multivariate linear regressions with dependent variable the citizenship acquisition rates (CAR) in the periods 2009–2013, 2014–2018, 2019–2023, and 2014–2023.

Periods and independent variables	Coefficients	Significance	Tolerance	VIF
2009–2013				
(Constant)	32.528	0.063		
MIPEX Citizenship Indicator	0.479	0.001	0.931	1.074
% of minors among foreigners (U18)	−1.290	0.142	0.885	1.130
% EU citizens among foreigners (UE)	−0.231	0.149	0.755	1.325
Refugees per 1,000 inhabitants (REF)	1.349	0.154	0.821	1.217
*R*^2^ (standard and adjusted in brackets)	0.666 (0.564)			
2014–2018				
(Constant)	10.070	0.539		
MIPEX Citizenship Indicator	0.512	0.001	0.988	1.012
% of minors among foreigners (U18)	0.108	0.889	0.910	1.099
% EU citizens among foreigners (UE)	−0.353	0.021	0.984	1.016
Refugees per 1,000 inhabitants (REF)	1.085	0.046	0.914	1.094
*R*^2^ (standard and adjusted in brackets)	0.668 (0.566)			
2019–2023				
(Constant)	−12.995	0.714		
MIPEX Citizenship Indicator	0.484	0.049	0.873	1.145
% of minors among foreigners (U18)	0.688	0.686	0.877	1.140
% EU citizens among foreigners (UE)	−0.177	0.485	0.938	1.066
Refugees per 1,000 inhabitants (REF)	0.938	0.230	0.911	1.097
*R*^2^ (standard and adjusted in brackets)	0.299 (0.084)			
2014–2023				
(Constant)	−3.765	0.871		
MIPEX Citizenship Indicator	0.508	0.008	0.942	1.062
% of minors among foreigners (U18)	0.375	0.738	0.911	1.098
% EU citizens among foreigners (UE)	−0.276	0.144	0.962	1.040
Refugees per 1,000 inhabitants (REF)	1.274	0.056	0.910	1.099
*R*^2^ (standard and adjusted in brackets)	0.515 (0.366)			

Sources: see Table 1.

The statistic units are equal to the 18 European selected countries.

The coefficient of the MIPEX Citizenship Indicator was consistently statistically significant (only at the 5% significance threshold in the period 2019–2023) and assumed values around 0.5. This indicates that, all other conditions being equal, an increase of one point in the value of the MIPEX Citizenship Indicator corresponded to a mean increase in the crude CAR of 0.5 citizenship acquisitions per 1,000 foreign residents. Among the other explanatory variables, the coefficients of the percentage of minors among foreigners were not significantly different from zero, while the coefficients of the percentage of EU citizens among foreigners were significant only in 2014–2018 with a negative sign as expected. The refugee impact presented significant regression coefficients both in the period 2014–2018 and in the decennial period (2014–2023), with a positive sign as expected (see the hypotheses).^15^

### 4.2 Specific and standardized CARs: the nexus with MIPEX and refugee impact

A key question concerns whether citizenship acquisition opportunities and propensities differ by citizenship status (EU vs. non-EU) and by age (persons under 20 years vs. adults). To address this, we calculated citizenship-specific CARs (for EU and non-EU) and age-specific CARs (for those under 20 years and those aged 20 and over). Figure 3 contains the values of the specific CARs for the three five-year periods (2009–2013, 2014–2018 and 2019–2023) and for the last decade (2014–2023).

**Figure 3:**
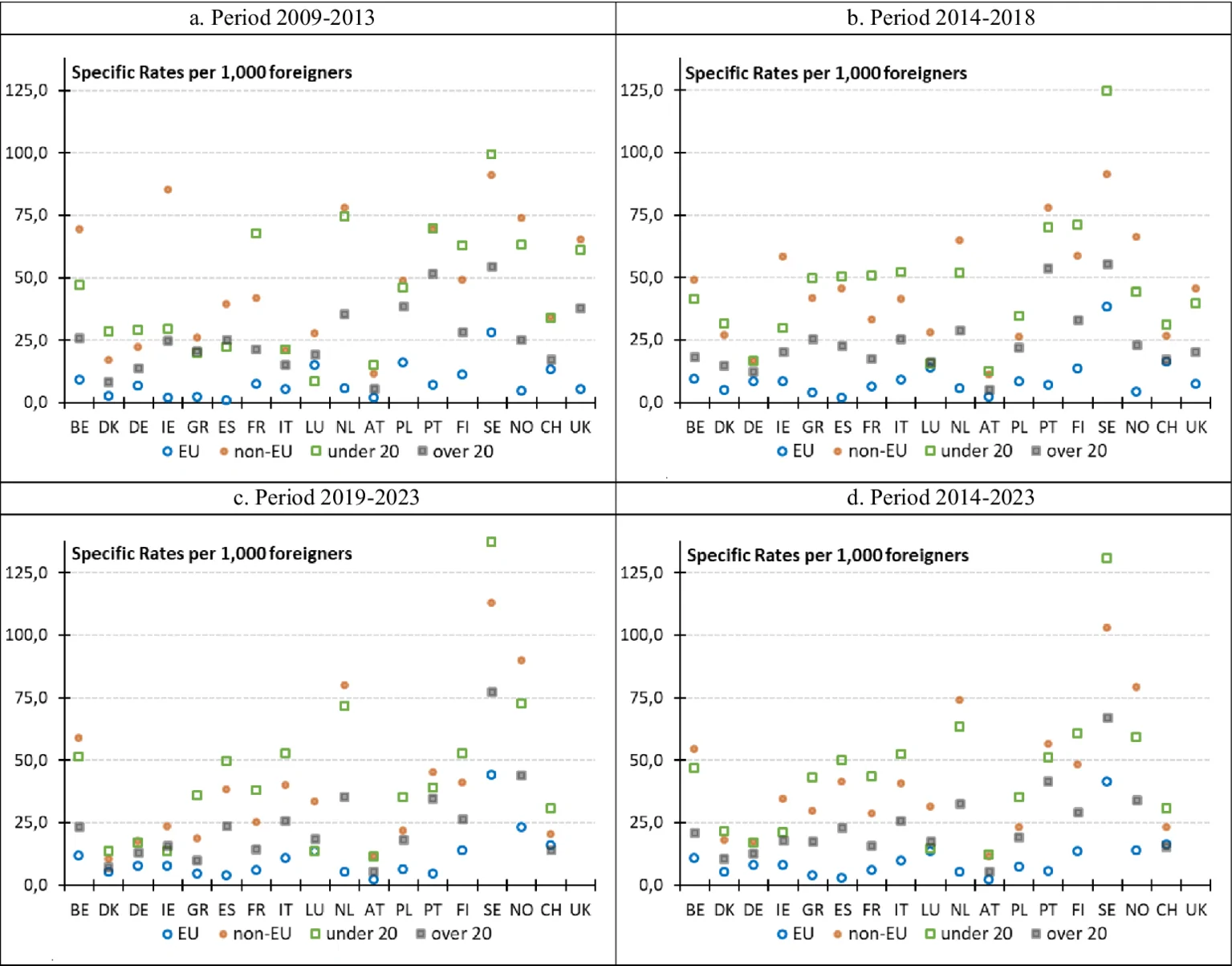
Citizenship acquisition rates by macro-area of foreign citizenship (EU and non-EU) and by large age groups (under 20 and over 20 years old) in 18 European selected countries^(a)^. Periods 2009–2013 **(a)**, 2014–2018 **(b)**, 2019–2023 **(c)**, and 2014–2023 **(d)** (rates per 1,000 foreigners of the corresponding category). ^(a)^See Table 1 for the correspondence between labels and countries. Sources: see Table 1.

The results clearly and uncontroversially show that foreigners with EU citizenship had lower propensity to acquire citizenship of the country of residence than those with non-EU citizenship. We observed this pattern in all countries considered and across all three five-year periods examined. The differential between the two groups (foreigners with EU citizenship and non-EU) in CAR values appears consistently wide, even in countries that are not Member States.

For age-specific CARs, the differences between the two main age groups considered appeared in some cases wide and almost always in favor of those under 20 years, albeit it was less pronounced than the ones observed between EU and non-EU foreigners. The pattern, however, was not uniform across countries and periods. In the period 2009–2013, Greece, Spain and Luxembourg showed higher CAR values for foreigners aged 20 and over than for those under 20. This pattern persisted across all periods in Luxembourg and also emerged in Ireland during the last five-year period (2019–2023). In all other countries, those under 20 years consistently had the highest CAR values, with particularly wide differences in France and Sweden. Moreover, in approximately half of the countries across all three five-year periods, the CAR values for those under 20 exceeded those for the entire set of non-EU foreigners.

In sum, these findings confirm that non-EU foreign nationals and younger foreigners (persons under 20 years, many of whom are second-generation immigrants) have greater propensities and/or opportunities to become citizens.

The question now is whether cross-country differences in the values of the four specific CARs are related to both the level of inclusiveness of citizenship legislation (measured through the MIPEX Citizenship Indicator) and a generally inclusive approach toward migrants (expressed by refugee impact on the overall population). To address this, as mentioned in Section 3.3, we estimated 20 multiple linear regression models (Table 3), with five dependent variables for each of the four periods (the three five-year periods and the decennial period). The five dependent variables were the four specific CARs plus the sex-age-citizenship-standardized CAR (SCAR), which controls for compositional differences in sex, age, and citizenship status across countries.

**Table 3:** Results of multivariate linear regressions with dependent variable the citizenship acquisition rates (CAR) by distinct categories and sex, age and citizenship standardized CAR^(a)^ in the periods 2009–2013, 2014–2018, 2019–2023, and 2014–2023.

Periods and independent variables	Dependent variable: citizenship acquisition rate (CAR) of									
	EU citizens		Non-EU citizens		Under 20 years		Over 20 years		Standardized CAR	
	Coeff.	Sign.	Coeff.	Sign.	Coeff.	Sign.	Coeff.	Sign.	Coeff.	Sign.
2009–2013										
Constant	−1.304	0.755	−4.286	0.718	−6.108	0.633	4.412	0.562	−2.862	0.696
MIPEX^(c)^	0.111	0.116	0.913	0.000	0.795	0.001	0.415	0.004	0.607	0.000
REF^(d)^	1.051	0.046	1.531	0.284	2.682	0.090	0.022	0.981	1.231	0.168
*R*^2^ adjusted	0.241		0.567		0.489		0.364		0.611	
2014–2018										
Constant	−7.830	0.053	−1.588	0.898	−8.849	0.555	−1.788	0.819	−3.470	0.637
MIPEX^(c)^	0.213	0.003	0.766	0.002	0.801	0.004	0.422	0.004	0.546	0.000
REF^(d)^	1.092	0.000	1.044	0.180	2.149	0.030	0.585	0.233	0.956	0.047
*R*^2^ adjusted	0.647		0.456		0.450		0.377		0.574	
2019–2023^(b)^										
Constant	−12.449	0.092	−4.213	0.000	0.600	0.983	−8.719	0.531	−7.648	0.658
MIPEX^(c)^	0.226	0.026	0.604	0.866	0.511	0.173	0.429	0.030	0.456	0.059
REF^(d)^	0.996	0.008	1.078	0.083	1.342	0.306	0.844	0.204	1.050	0.205
*R*^2^ adjusted	0.413		0.103		0.041		0.221		0.162	
2014–2023^(b)^										
Constant	−12.448	0.013	−5.442	0.767	−8.034	0.701	−7.211	0.467	−8.076	0.500
MIPEX^(c)^	0.235	0.002	0.664	0.023	0.664	0.042	0.434	0.008	0.495	0.011
REF^(d)^	1.209	0.000	1.420	0.172	2.003	0.097	0.938	0.098	1.305	0.061
*R*^2^ adjusted	0.671		0.261		0.242		0.371		0.366	

^(a)^Direct standardization by sex, age (under 20 and 20 and over) and nationality (EU and non-EU foreign nationals). ^(b)^Excluding the United Kingdom for the periods 2019–2023 and 2014–2023. The statistic units are equal to the 18 European selected countries. ^(c)^MIPEX Citizenship Indicator. ^(d)^Refugees per 1,000 inhabitants.

Sources: see Table 1.

In 2009–2013, the two predictors showed the greatest explanatory capacity when the dependent variable was the sex-age-citizenship-standardized CAR (adjusted *R*^2^ = 0.611) and the CAR for non-EU citizens (adjusted *R*^2^ = 0.567). In the following five-year period, the explanatory capacity of the two independent variables remained high when the dependent variable was the sex-age-citizenship-standardized CAR (*R*^2^ adjusted = 0.574) but was greater for the CAR for EU citizens (*R*^2^ adjusted = 0.647). In both the third five-year period and the decennial period, the MIPEX Citizenship Indicator and refugee impact showed the greatest explanatory capacity when the dependent variable was the CAR for EU citizens.

The higher explanatory capacity of the model when the dependent variable was the CAR for EU citizens depended on the importance assumed by the refugee impact indicator among the independent variables. Indeed, across all periods considered, only for CARs for EU citizens did the positive coefficient relating to the refugee impact appear particularly large and significant. The meaning of this relationship is not immediately straightforward. The refugee impact indicator, which is at least a signal of the country’s openness toward forced migration (see Section 2.4), could also have functioned as a proxy for the presence of generally more or less favorable conditions for immigrants, sufficient to encourage even EU nationals themselves to acquire citizenship of the country. These considerations also applied when the dependent variable was the CAR for those under 20 years and the coefficient relating to the refugee impact indicator was statistically different from zero and particularly high (first two five-year periods and decennial period).

When the dependent variable was the CAR for non-EU citizens, the coefficient relating to the MIPEX Citizenship Indicator was statistically different from zero in three of the four regressions (the exception being the last five-year period) and particularly large—larger than when the dependent variable was the CAR for EU citizens. Moreover, the coefficient of the MIPEX Citizenship Indicator was positive and statistically significant in all regressions where the dependent variable was the CAR for foreigners aged 20 and over. In these regressions, the regression coefficient of the refugee impact indicator was never significant (only in one regression it was significant at the 10% threshold).

Finally, we examined the relationship between the two predictors and the standardized rate. Specifically, we aimed to verify whether the two independent variables capture a larger share of the variance of the dependent variable when this is calculated as a standardized rather than as a crude rate. At least for the first two five-year periods, models using the SCAR as the dependent variable showed superior performance compared to those using the crude CAR. The two independent variables exhibited greater explanatory capacity, and their coefficients aligned with expected values. For the third five-year period, the capacity of the two predictors to capture variability SCAR variability was greater than that of capturing crude CAR variability, though it remained limited. While the explanatory capacity of the two independent variables was confirmed, additional indicators should probably be considered to explain a larger share of the variation in citizenship acquisition rates in the selected European countries, at least for the most recent period. Overall, the SCAR appears a more appropriate measure for cross-country comparisons of citizenship acquisition intensity.^16^

## 5 Discussion and conclusion

This study examined the relationship between citizenship policy inclusiveness and citizenship acquisition outcomes across eighteen European countries over the period 2009–2023. By combining a synthetic policy indicator—the MIPEX Citizenship Indicator—and multiple measures of acquisition intensity, the analysis confirms that formal legislative inclusiveness matters, but only as part of a broader and more complex picture (Janoski, 2010; Reichel, 2011; Vink, 2017; Huddleston and Falcke, 2020).

Despite the limitations already acknowledged in Section 2 and for the reasons extensively discussed therein, the study adopted the MIPEX Citizenship Indicator as a synthetic measure of the inclusiveness of national citizenship regimes.

The empirical analysis in Section 4 calculated crude, specific, and standardized CARs and examined the relationship between citizenship outputs (measured through the MIPEX Citizenship Indicator) and outcomes (measured through CARs). Overall, the results confirmed that the MIPEX Citizenship Indicator—at least for the first two of the three five-year periods examined—is a meaningful predictor of the crude CAR, thereby supporting the expected positive relationship between policy outputs and outcomes. However, the model’s explanatory power was only moderate and weaker in the most recent period, evidencing—on the one hand—the already discussed limits of the MIPEX Citizenship Indicator, showing that the dimensions of citizenship regimes not included in this Indicator, such as the full range of implementation practices, discretionary thresholds, procedural costs, or category-specific facilitations are not marginal, but may shape access to citizenship in practice, and—on the other—that additional indicators should be considered as independent variables. For this reason, the analysis took into account three compositional variables: the proportion of EU nationals, the proportion of persons under 20, and refugee impact on the total population, and the results exhibit that they also matter in their own right. The expected negative relationship between crude CAR and the proportion of EU nationals among foreign residents was confirmed, although the coefficient achieved statistical significance only in one of the regressions. By contrast, the proportion of persons under 20 among foreign residents did not show a statistically significant positive influence on the crude CAR. On the other hand, refugee impact on the relative frequency of citizenship acquisitions was mostly confirmed as positive, with the regression coefficient attaining statistical significance in two of the four regressions. The intensity of citizenship acquisition thus appears to be associated not only with formal policy inclusiveness but also with the composition of the foreign resident population and with the country’s broader openness towards non-citizens (as reflected in the importance accorded to refugees).

The citizenship-specific (EU and non-EU) and age-specific (under 20 and 20 and over) CARs further confirmed that persons under 20 generally exhibit higher propensities and/or opportunities to acquire citizenship than persons aged 20 and over, whereas EU nationals tend to acquire country of residence citizenship less often, presumably because they have weaker incentives to do so. However, not all regression results conformed to expectations. It was hypothesized that the MIPEX Citizenship Indicator would exhibit greater predictive power with respect to the CARs of non-EU citizens and persons aged 20 and over. In the first two five-year periods, the MIPEX Citizenship Indicator had stronger explanatory power when the dependent variable was the CAR of non-EU citizens, thereby confirming the hypothesis. By contrast, this pattern reversed in 2019–2023, when the coefficient of the MIPEX Citizenship Indicator was statistically significant only when the dependent variable was the CAR of EU citizens. Furthermore, although the coefficient remained consistently significant when the dependent variable was the CAR of persons aged 20 and over, its value was consistently higher when the dependent variable was the CAR of persons under 20. Finally, the hypothesis that rates standardized by sex, citizenship, and age would increase the explanatory power of the predictors (MIPEX Citizenship Indicator and refugee impact) was largely confirmed: the explanatory capacity of the two independent variables modestly improved, indicating that controlling for sex, age, and citizenship composition reduces compositional bias and yields a more comparable measure of acquisition intensity across countries. In sum, the findings suggest that legislation matters for effective access to citizenship in the countries examined, although the measures employed remain imperfect and it is likely that other explanatory factors warrant attention, particularly for the most recent five-year period.

This contribution nonetheless presents some limitations. First, the absence of longitudinal data tracking specific cohorts of first- and second-generation trajectories constrained the analytical approach to cross-sectional aggregate comparisons rather than more refined dynamic specifications. Second, the necessity of employing specific and synthetic cross-sectional CARs that control for the differential characteristics of foreign resident populations across countries. This limitation has been partially overcome through the development of citizenship-specific and age-specific CARs, and the proposed SCAR controlling for compositional differences. Third, the MIPEX Citizenship Indicator does not capture many of the legal and institutional details of citizenship regimes, nor administrative implementation practices that shape *de facto* access to citizenship. Finally, substantial cross-national variance in CARs persists even after controlling for formal policy inclusiveness and compositional factors. This suggests that additional dimensions—such as labor market integration opportunities, educational pathways, social networks, and subjective perceptions of belonging—shape naturalization propensities. These factors merit investigation, but they extend beyond the institutional and demographic focus of this study, which deliberately concentrates on the relationship between formal legal frameworks and acquisition outcomes.

Notwithstanding these considerations, the study contributes to the empirical literature by proposing methodological innovations through citizenship-specific and age-specific CARs and introducing the SCAR as a standardized measure that controls for compositional differences. Additionally, the study empirically substantiates the linkage between policy outputs and outcomes across multiple periods and population subgroups, while also revealing temporal and group-specific variations that enrich our understanding of how formal citizenship regimes translate into actual acquisition patterns.

To conclude, taken together, the findings of this analysis point to a broader implication: citizenship acquisition is shaped not only by formal policy inclusiveness, but also by the interaction between policy design, population composition, and institutional context. MIPEX remains useful precisely because it allows for structured comparison across countries and over time. Yet its explanatory power is enhanced when it is interpreted as one component of a broader analytical framework rather than as a stand-alone measure of citizenship access. In this sense, the paper does not suggest that MIPEX should be replaced. Rather, it shows that its results need to be read in light of the structural and compositional factors that shape who naturalizes, when, and under what conditions.

More generally, the study confirms that standardized citizenship policy indicators retain significant value for comparative research, but also that they should not be treated as exhaustive representations of citizenship regimes. Where the relationship between policy outputs and naturalization outcomes is weaker or unstable, the explanation may reside in both the inherent limits of the indicators and in the gap between formal rules and their practical operation. Future research should therefore combine quantitative policy measures with more fine-grained qualitative information on administrative implementation, discretionary practices, and category-specific access routes, particularly where the evidence suggests differentiated acquisition patterns across sub-populations defined by age and citizenship status.

The analytical framework and measurement strategies developed in this study provide tools that may serve as a basis for more differentiated measures of citizenship acquisition outcomes (CARs and SCARs) and, in future work, for the development of more targeted policy indices that better reflect the differentiated acquisition pathways across sub-populations.

## Data Availability

Publicly available datasets were analyzed in this study. This data can be found at: Eurostat (https://ec.europa.eu/eurostat/data/database); MIPEX (https://www.mipex.eu/); UNHCR Refugee Data Finder (https://www.unhcr.org/refugee-statistics); and the GLOBALCIT Citizenship Law Dataset, including the modes of acquisition (https://globalcit.eu/modes-acquisition-citizenship/) and loss of citizenship (https://globalcit.eu/modes-loss-citizenship/).

## Footnotes

1 Citizenship can be viewed as both a tool for integration and a reward for integration, reflecting debates on whether it should function as a means or culmination of inclusion.

2 The term ‘ordinary naturalization’ is used to refer to the general rules defining access to citizenship after birth.

3 While it would be possible to construct a synthetic indicator based on the GLOBALCIT Citizenship Law Dataset, any such measure would necessarily be limited to the 2020–2024 period, the only period currently covered by the dataset.

4 For a fuller discussion of States’ capability to grant protection, see Forte et al. (2025). However, it should be noted that refugee impact, while capturing the volume of asylum applications and recognized refugees, does not capture the nationality profile of applicants, the filtering effect of Dublin procedures, or the fact that asylum adjudication is formally carried out by independent bodies in many countries. It may therefore reflect both policy orientation, broader institutional capacity, and governmental receptivity.

5 This consideration could be partly revised in the last period considered in the analysis considering the role played by geographic proximity in the distribution of Ukrainian refugees across EU Member States, as illustrated by the importance of Poland.

6 EUROSTAT refers to Regulation (EC) No 862/2007 (European Parliament and Council of the European Union, 2007) for the definitions used and states that, for citizenship acquisition data, the relevant category comprises ‘persons having their usual residence in the territory of the Member State and having acquired during the reference year the citizenship of the Member State and having formerly held the citizenship of another Member State or a third country or having formerly been stateless […]’. This strengthened our view that the citizenship acquisition rate is a reasonably appropriate measure.

7 According to EUROSTAT data (migr_pop1ctz), on 1 January 2016—the middle of the considered period – the foreign resident population in the eighteen countries ranged from near 8,652,000 in Germany to near 150,000 in Poland in absolute terms, and from 46.7% of the total population in Luxembourg to 0.3% in Poland in relative terms.

8 Specifically, Norway’s proximity to the EU migration and asylum governance system is manifested by its participation in the EEA and Schengen Area, while Switzerland’s alignment is managed through a comprehensive network of bilateral agreements with the EU, which also include participation in the Schengen and Dublin acquis.

9 By contrast, breakdowns by nationality are missing or incomplete most notably for the United Kingdom (2009–2019, when cross-tabulated with age), France (2009–2013, and 2014–2024 when cross-tabulated with age), Greece (2009–2015 and 2022–2024), and Poland (2009–2015 and 2021–2024). For 2012, Sweden’s age breakdown was incorrect, while Switzerland’s was missing.

10 For the United Kingdom, EUROSTAT statistics are available through 2019, thereafter, since it was only partially possible to supplement them, disaggregated data by sex, age and citizenship are unavailable.

11 We use the term ‘intensity’ to denote the relative frequency of citizenship acquisitions—the ratio of acquisitions to average foreign population. ‘Relative frequency’ is sometimes used as a synonym to emphasize the relational and derivative nature of this measure.

12 When referring to existing literature, we use the term ‘naturalization rates’, while from Section 3 onwards we adopt ‘CAR’ for the reasons outlined above.

13 Given the small number of statistical units (18 countries), we conducted robustness checks by repeating the same regressions indicated in Section 3.4 for all 18 countries across the three five-year periods (pooled model). In this way, we worked with 54 statistical units when the dependent variable was crude CAR and with 53 statistical units when the dependent variables were the specific CARs or SCARs (due to missing UK data in the last period). The results of these analyses are summarized below in two supplementary notes.

14 Here and throughout this article, we do not discuss the values disaggregated by sex as the correlation between the vector of the male and female rates is extremely high across all the 15 years considered (from 0.94 to 0.98), with broadly similar values.

15 The pooled model (54 statistical units corresponding to the product of the 18 countries in the three five-year periods), with an adjusted *R* of just under 0.4, confirms results from the main analysis just described. Among considered predictors, the MIPEX citizenship indicator emerges as the strongest predictor of crude CAR, with a coefficient value close to 0.5 and highly significant. Among remaining explanatory variables, only the percentage of EU citizens among foreigners and the impact of refugees on the total population show regression coefficients significantly different from zero at a 5% significance level. Adding two dummy variables to take into account the three five-year period yields unchanged substantive results, with only the 2019–2023 coefficient statistically significant.

16 The results just described are essentially confirmed by the pooled analysis (*N* = 53 statistical units). The coefficient of the MIPEX Citizenship Indicator remains highly significant, especially when the dependent variables are CAR for non-EU citizens, CAR for foreigners aged 20 and over, or SCAR. Furthermore, the variability explained by the MIPEX Citizenship Indicator and refugee impact on total population appears greater for SCAR than crude CAR. When two dummy variables are added to account for the three five-year periods, results do not change, confirming differences between the first two periods and the third.
